# Heavy metals and eggshell coloration in House Sparrow (*Passer domesticus*) eggshells across the Eastern United States

**DOI:** 10.1371/journal.pone.0336122

**Published:** 2026-02-25

**Authors:** Suzanne Hartley, Caren Cooper, Mariah Patton, Chris Hawn, Kathryn Jewell, Aubrey Wiggins, Memuna Khan, Daniel Hanley

**Affiliations:** 1 Department of Forestry and Environmental Resources, North Carolina State University, Raleigh, North Carolina, United States of America; 2 North Carolina Museum of Natural Sciences, Raleigh, North Carolina, United States of America; 3 Department of Biology, University of New Mexico, Albuquerque, New Mexico, United States of America; 4 North Carolina Environmental Justice Network, Raleigh, North Carolina, United States of America; 5 Department of Biology, Ripon College, Ripon, Wisconsin, United States of America; 6 Department of Biology, George Mason University, Fairfax, Virginia, United States of America; University of Akron, UNITED STATES OF AMERICA

## Abstract

House Sparrows (*Passer domesticus*) may serve as suitable species to monitor persistent environmental contaminants, like heavy metals, that pose serious health risks to humans and wildlife alike. Avian eggshells have the potential to be used as indicators of environmental contaminants since these can alter many chemical pathways, including those involved in forming and depositing avian eggshell pigmentation. Here we tested House Sparrow eggs for heavy metals and examined whether eggshell pigmentation predicted heavy metal concentrations. As part of the citizen science project, Sparrow Swap, volunteers across the United States collected 536 clutches, totaling 2,182 House Sparrow eggs. We then tested whether metal concentrations were predicted by coloration and speckling of these eggs or eggshell thickness and calcium concentration. We found that metals, including As, Se, Cd, Cu, and Pb, were present in detectable levels in House Sparrow eggs from across the country. Although eggshell characteristics were not strong predictors of metal concentrations, metal concentrations in the eggshells were higher than expected and warrant further investigation.

## Introduction

As humans continue to modify the environment, it is increasingly important to find organisms that can serve as indicators for the health of wildlife and the environment. Such bioindicators should be sensitive to environmental contaminants, easy to observe, widely distributed, well studied, and of interest to the public to make these organisms ideal bioindicators [1]. Although a range of several avian tissues (e.g., blood, feathers, excrement, and egg contents; [1]) have been used to monitor harmful environmental contaminants (e.g., heavy metals, organochlorines, and pyrethroids), avian eggshells are particularly valuable as bioindicators as they can serve as a sink for excreted heavy metals [2] and can be monitored non-destructively. In addition, monitoring trace elements in avian eggshells is useful to monitor potential impacts on avian reproductive success. Trace elements such as selenium and copper can impair the quality of eggshells by decreasing hatching success [3] through reducing eggshell thickness, size, and hatchability [4].

**Fig 1:**
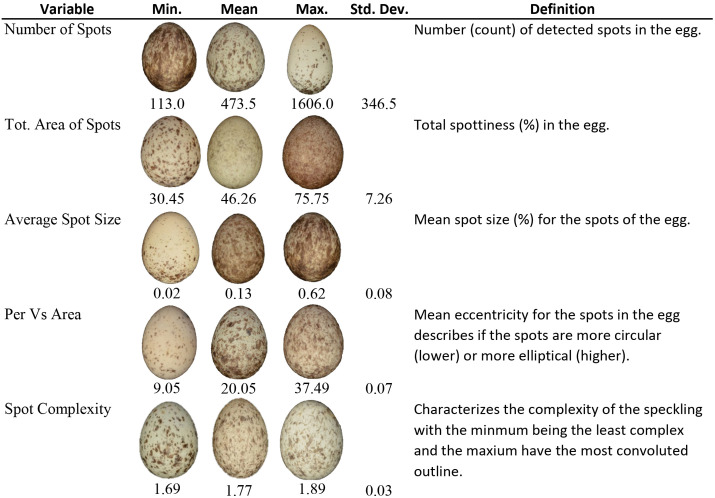
Summary of SpotEgg Results. The minimum (min.), mean, maximum (max.), standard deviation (std. dev.), and definition for each variable computed from SpotEgg. Some eggs represented the min. or max. for more than one variable.

**Fig 2:**
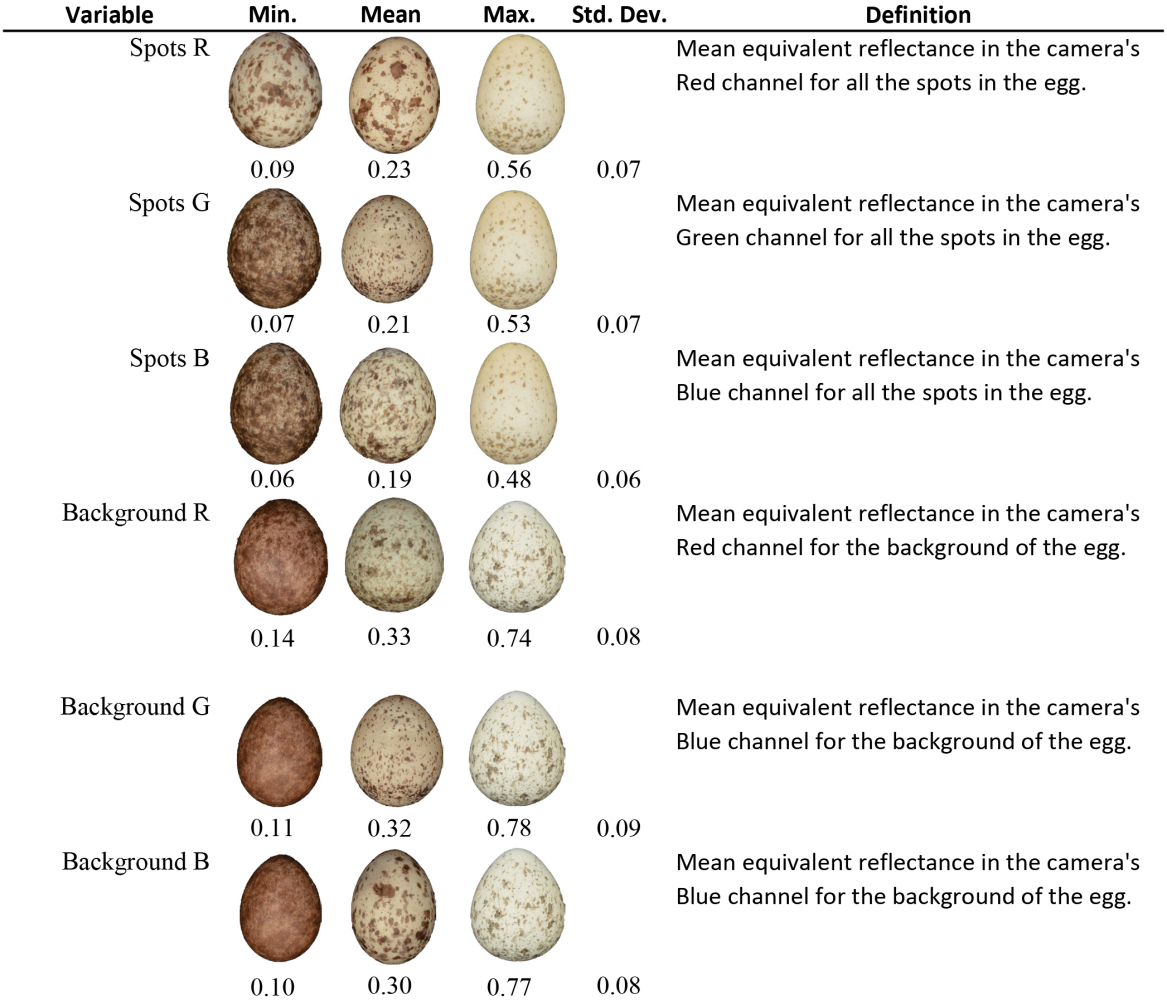
Summary of SpotEgg Results (Continued). Summary of SpotEgg Results (Continued) The minimum (min.), mean, maximum (max.), standard deviation (std. dev.), and definition for each variable computed from SpotEgg. Some eggs represented the min. or max. for more than one variable.

For eggshells to be useful as a non-destructive bioindicator, it is important to select a suitable bird species. To provide adequate resolution and spatial coverage of environmental contaminants, a bioindicator species should have a large distribution but relatively small home ranges. Past studies have examined metal contaminants in eggshells; however, many of these studies focused on migratory species, which can make determining the source of the contaminants difficult [5,6]. For example, it may be difficult to determine if the contaminants detected occurred in wintering grounds, breeding grounds, or from a stopover location without repeated testing of individual birds before and after exposure. By contrast, House Sparrows (*Passer domesticus*) are great bioindicator candidates because they are ubiquitous, commensal with humans, non-migratory, have a small home range (<8 hectares), and possess variable eggshell colors and patterns (Figs 1 and 2) [7,8]. Moreover, House Sparrow populations have been introduced outside their native range and are found across the world. This study focuses on the United States, where the House Sparrow is an invasive species and is not federally protected by the Migratory Bird Treaty Act of 1918 [9,10].

Environmental contaminants, such as heavy metals, can alter the heme biosynthesis pathway that gives rise to the four pyrrole pigments [11–14] responsible for generating the diversity of colors found in avian eggshells [15]: biliverdin (blue), protoporphyrin (reddish-brown), tetrapyrrolic bilirubin (yellow-brown), and tripyrrolic uroerythrin (red-orange) [16,17]. Not only can contaminants alter which pigments are up- or down-regulated, thereby impacting eggshell ground color, but they can also interfere with how and where pigments are deposited in eggshells, impacting the concentration and density of pigmented spots. For example, the structural-function hypothesis proposes that because protoporphyrin shares a carrier protein with calcium, the pigment is deposited where calcium is limited and thus strengthens thinner areas of the eggshell [18,19]. Thus, if heavy metals disrupt the ionic uptake of calcium, they could alter the creation and composition of eggshells [20]. Gosler and Wilkin [21] found that decreasing calcium availability was correlated with decreased eggshell thickness and decreased eggshell pigment spread, suggesting eggshell coloration could be a useful non-destructive bioindicator.

Here, we collected House Sparrow eggs, which would otherwise have been discarded, from around the continental United States to determine whether the colors and patterns on these eggs could be used to monitor heavy metals in the environment that are mobilized into food webs and whether eggshell characteristics can predict the concentrations of heavy metals. To do so, we quantified eggshell characteristics of coloration, speckling, thickness, and calcium concentration and extracted the concentration of heavy metals (Cu, Pb, Cd) and metalloids (Se and As) found within these House Sparrow eggs (hereafter metals). Metals were selected for their potential to impact eggshell characteristics [22] and impacts on the health of wildlife and humans [23–25]. There may be other metals not included in this study that may have impacts on eggshell characteristics or the health of wildlife and humans. We expect that eggs with higher metal content will have thinner shells, more speckling and reduced calcium concentrations. However, since the spot complexity could be a better indicator than the color of speckling, we also might expect that metals could influence the complexity of eggshell speckling. In addition, we expect the blue-green color of the eggshells to decrease with increasing concentrations of metals as predicted if environmental contaminants act as stressors [8,11,12].

## Materials and methods

### Egg collection

**Fig 3:**
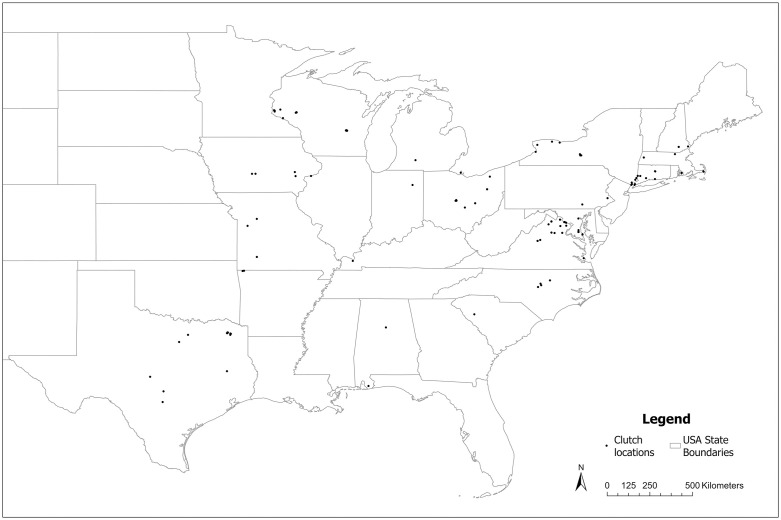
Map of clutch locations. House Sparrow clutches were collected from nestboxes across the eastern half United States (n = 431). Basemap from U.S. Census Bureau, “cb_2018_us_state_5m”, TIGER/Line Shapefiles, 2018, https://www.census.gov/geographies/mapping-files/time-series/geo/carto-boundary-file.html/, accessed on March 23, 2025.

House Sparrow eggs were collected via Sparrow Swap, a citizen science project in which volunteers sent House Sparrow eggs to scientists at the North Carolina Museum of Natural Sciences. The project was open to anyone in the United States from March 1, 2016 until July 12, 2018. Volunteers were presented with an informed consent form and consented to participation by selecting “yes” on the form prior to joining the project, which was hosted via Scistarter, an online citizen science platform. As a non-native species, House Sparrows are not protected in the United States, and no permits were needed for egg collection. If volunteers were not the property owners, participants were responsible for ensuring they had permission to collect eggs at the nest location. We received approval for our protocol (#11949) from North Carolina State University’s Internal Review Board. No Institutional Animal Care and Use Committee approval was needed since eggs are not considered to be vertebrates. During the breeding season of these years, 431 House Sparrow clutches (over 2,182 eggs) were opportunistically collected across the eastern half of the United States (Fig 3) by volunteers who encountered House Sparrow nests in nestboxes established to attract native songbirds such as bluebirds (*Sialia sialis, Sialia mexicana, Sialia currucoides*) and Tree Swallow (*Trachycineta bicolor*).

Participants were instructed to wait until a House Sparrow had completed their clutches before removing their eggs. Once collected, volunteers placed the eggs in a refrigerator for at least 24 hours to stop any further development of the embryo. Volunteers carefully packaged the eggs per Sparrow Swap protocol and mailed the eggs, which were unpacked and cataloged shortly after they arrived at the museum. In addition to a unique catalog number that each clutch was assigned, each egg in a clutch was also assigned a specific letter (A-G).

### Quantification of colors and patterns

**Fig 4:**
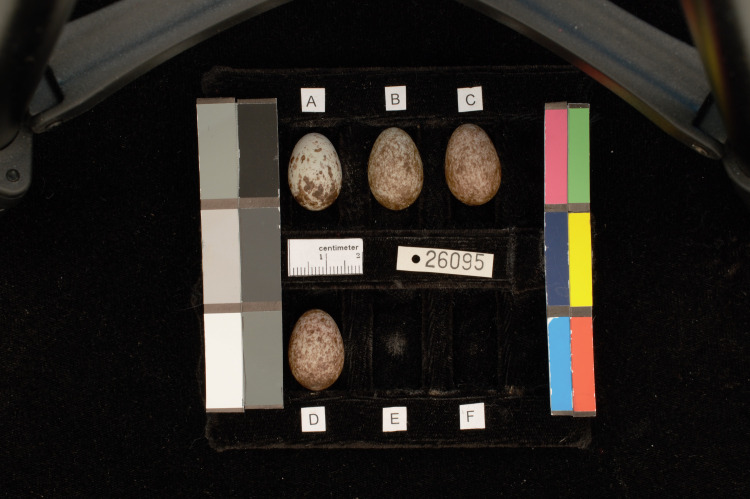
Example of a standardized clutch photograph. Photographs were taken by clutch, with each egg assigned a letter. The photographs included color charts and a ruler.

We photographed each of the 1,462 eggs that arrived in good condition following a standardized protocol using a NIKON 3200 digital SLR camera (Aperture: F16, Shutter speed: 1/1.6). Photographs were taken in a RAW, lossless file format (.NEF). In each clutch photograph, we included a scale and six grayscale color patches (DKC-Pro Color Chart) of known spectral reflectance (Fig 4).

**Fig 5:**
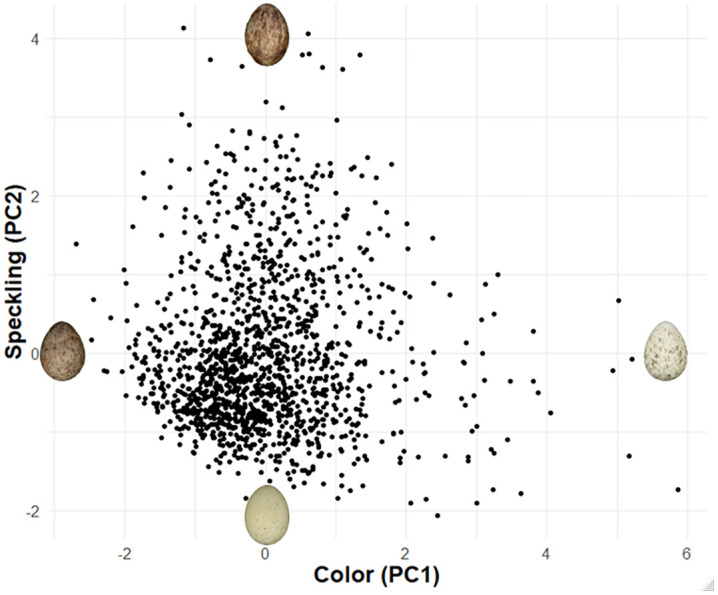
Principal Component Analysis Graph. Distribution of eggs (n = 1,426) across the two principal components representing color (PC1) and speckling (PC2) of the eggs.

We used SpotEgg, an image processing tool created by Gómez and Liñán-Cembrano [26], to quantify the color and speckling of each House Sparrow egg. SpotEgg equalizes the RAW (.NEF) images using DCRAW [27], a tool used for processing raw image files. SpotEgg then employs MatLab software to detect and quantify the area (%) of speckling on each egg and the color (as RGB) values of each spot (Figs 1 and 2). To reduce the highly correlated variables and their interactions, we conducted a principal component analysis (PCA) with Varimax (orthogonal) rotation of the 10 variables relating to eggshell color and speckling using the psych package in R [28]. Spot size and total area of spots were transformed (1/x) for the PCA to make the scaling similar to the other variables included in the PCA. The varimax rotation allowed for easier interpretability in subsequent analyses. The PCA on the color and speckling variables yielded two principal components (PCs) explaining 90% of the variance between eggs. The first principal component (PC1) mainly explained background and spot color Table 1. Eggs with positive loading values were lighter in both background and spot color than eggs with negative PC1 loadings. The second principal component (PC2) represented the amount, size, and shape of the eggshell’s speckling Table 1. Eggs with a high PC2 score had more spots that were larger and more irregularly shaped than eggs with lower PC2 scores, which had fewer, smaller more circular spots. Together, PC1 and PC2 explained 54% and 36% of the variance, respectively (Fig 5).

**Table 1:** PCA Results for Eggshell Characteristics.

Variable	PC1	PC2
Spots R	**0.93**	0.32
Spots G	**0.92**	0.33
Spots B	**0.91**	0.34
Background R	**0.93**	0.31
Background G	**0.92**	0.33
Background B	**0.93**	0.32
Number of Spots	0.30	**0.92**
Per vs Area	−0.19	**−0.83**
Average Spot Size (1/x)	0.37	**0.96**
Total Area of Spots (1/x)	0.31	**0.74**
Proportion of Variation Explained	0.54	0.36
Cumulative Proportion	0.54	0.90

Loadings for each of the 10 variables included in the principal component analysis with a variamax rotation. PC1 has strong positive loadings (bolded) for spots R, spots G, spots B, background R, background G, and background B. PC2 has strong positive loadings (bolded) for number of spots, per vs area, average spot size, and total area of spots.

After the eggs were photographed, the eggs were cut using a Dremel 200 Series rotary tool, and the eggshells were rinsed with deionized water and air-dried at room temperature for at least 36 hours. The blunt end of the egg and an approximately 8 x 8 mm piece from the equator of the egg were reserved for thickness measurements. Eggshell thickness was measured using a low-force Mitutoyo micrometer (Digimatic Micrometers Series 227) with the force set to 0.05 N and measured to the nearest 0.001 mm, similar to Igic et al. [29]. Three measurements were taken from the blunt end of the egg, and another three thickness measurements were taken from the piece of the egg taken from the egg’s equator. The thickness of eggshells ranged from a minimum of 0.090 mm to 0.165 mm, with a mean of 0.118 mm and a standard deviation of ±0.012 mm. From the 1,426 eggs analyzed for eggshell characteristics, one hundred eggs from complete clutches were subsampled for metal analysis. In addition, we only selected eggs that were in the early development stage since calcium and metal concentrations can change in the eggshell over time as the embryo develops [30]. To classify eggs into developmental stages we halved eggs and classified them as early if the yolk was still intact and no red blood vessels were visible, middle if red blood vessels were visible and the embryo was visible but lacked distinct morphology, and late if the embryo had visible morphological traits (e.g., beak and eyes). To represent the entire clutch, we chose one egg from each of the 431 clutches that had PC1 and PC2 scores closest to their clutch average. Out of the 431 average eggs selected, we analyzed 100 eggshells for metal concentrations. To capture the full range of the color and speckling, we chose 20 eggs from the maximum of PC1, 20 eggs from the minimum of PC1, 20 eggs from the maximum of PC2, 20 from the minimum of PC2, and 20 eggs from the middle of PC1 and PC2.

### Quantification of egg contaminants

Eggshells selected for metal concentration quantification were rinsed with acetone, followed by a rinse with distilled water, and then air-dried again. Eggshells were homogenized using a mortar and pestle, rinsing with acetone after each sample. Eggshell samples were then sent to the Environmental and Agricultural Testing Service (EATS) at North Carolina State University for sample digestion and elemental testing of Ca, Cu, Se, As, Pb, and Cd. Sample preparation and testing were similar to Hargitai et al. [22]. At the EATS laboratory, HNO3 was added to the dried eggshell samples to digest the eggshells into a solution for analysis. Calcium concentrations were analyzed using a Perkin Elmer ICP-Optical Emission Spectrometer Model 8000. To quantify metals at lower concentrations, the samples were spiked with 0.5g/L of metals (As, Cu, Pb, Se, Cd). This concentration was then subtracted from the final reporting. Metals (As, Cu, Pb, Se, Cd) were analyzed using a Perkin Elmer Elan DRCII ICP- Mass Spectrometer. Spiked solutions were also periodically analyzed with samples to more accurately determine the concentration of the spike solution over sample run times. The practical quantitation limit (PQL), the limit at which the elements can be accurately quantified, and the method detection limit (MDL), the threshold where elements can be detected, varied by element. For eggshells with metal concentrations below the MDL, half the MDL limit was used. For eggshells above the MDL but below the PQL, the mean of the MDL and PQL was used.

Before conducting any metal analyses, we determined if there were correlations between metal concentrations. Because distributions of trace elements did not follow a normal distribution, we tested correlations between metals using Kendall’s tau (non-parametric) Table 2.

**Table 2:** Kendall correlation table for metals.

Kendall’s τ_a_	Ca	As	Cd	Cu	Pb	Se
**Ca**	*******					
**As**	−0.02 (*p* = 0.73)	*******				
**Cd**	0.06 (*p* = 0.42)	**0.30** **(*p* < 0.001)**	*******			
**Cu**	−0.13 (*p* = 0.06)	−0.11 (*p* = 0.12)	−0.09 (*p* = 0.23)	*******		
**Pb**	−0.09 (*p* = 0.21)	**−0.16** **(*p* = 0.02)**	−0.08 (*p* = 0.319)	**0.47** **(*p* < 0.001)**	*******	
**Se**	0.02 (*p* = 0.73)	−0.01 (*p* = 0.94)	0.02 (*p* = 0.78)	0.10 (*p* = 0.14)	−0.01 (*p* = 0.89)	*******

Values reported are *τ*_*a*_ with probability in parentheses. Significant findings are bolded.

We also conducted a PCA with the metal concentrations to determine if we could create one or two variables that we would consider as “contaminant load”. However, we found that the first principal component explained less than 20% of the variation, thus, we chose to test the metals individually. We found that thickness, color (PC1), and speckling (PC2) were uncorrelated with each other; the r varied from −0.10 to 0.10 (p > 0.30). Calcium was uncorrelated with color and speckling, the r varied from −0.20 to 0.20 (p > 0.10). Ca and thickness were negatively correlated with each other (r = −0.21, p = 0.04). To test whether eggshell characteristics were a potential indicator of contaminants we used a generalized linear model in the stats package of R [31] with metal concentrations (As, Cu, Pb, Se, Cd) as the response variable and thickness (mm), calcium concentration (%), color (PC1), speckling (PC2), latitude, longitude, and collection date (ordinal date) as predictor variables with interaction terms for collection date and color (PC1) and an interaction term for latitude and longitude. Because cadmium was found in only a small quantity of the eggshells sampled, we used a binomial generalized linear model with 1 designated to samples where quantifiable amounts of cadmium were detected and 0 for samples where there was not a measurable concentration of cadmium. To test whether metals were a potential indicator of protoporphyrin content, we used a generalized linear model in stats package of R [31] with spot complexity, which SpotEgg calculated as fractal dimension, as the response variable and metal concentrations and their interactions (As, Cu, PB, Cd, As) as the predictor variables. The Akaike Information Criterion (adjusted for small sample size) (AICc), delta AICc, and weight of each model were determined using the dredge function and averaged using the model.avg function, both found in the MuMIn package of R [32]. The average model assumed that all variables were included in every model. In models where the variable was considered a weak predictor, the corresponding coefficient was set to zero.

## Results and discussion

### Elemental concentrations and thickness

**Fig 6:**
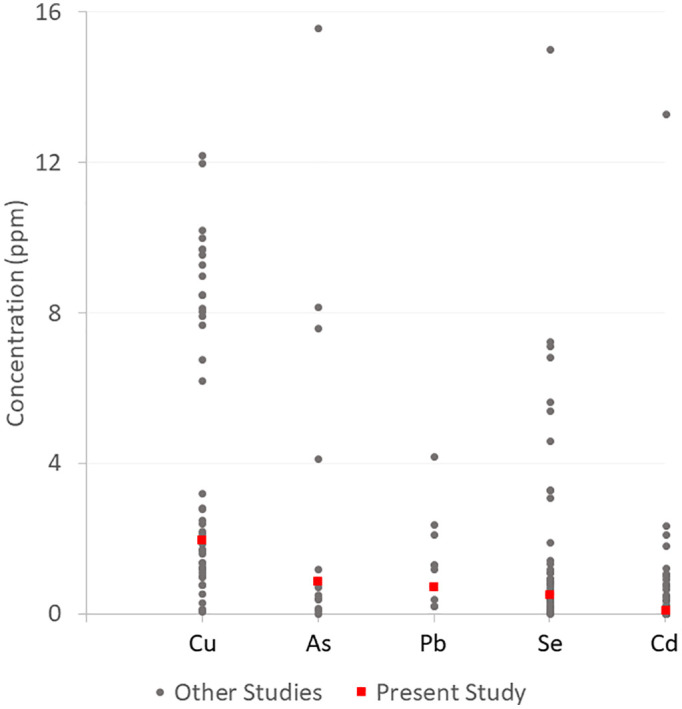
Comparison of mean concentrations of metals in House Sparrow eggshells to other studies. The concentrations of metals in House Sparrow eggshells fall within the range of concentrations found in the eggshells of various bird species.

Since eggshells are mostly composed of calcium carbonate, calcium concentrations ranged from 30.86% to 37.47%, with an average of 34.69% (±1.60). For the remaining elements, only 27 of the 100 eggshells had all 5 metals present at detectable levels. Most eggshells (n = 46) had a combination of 4 of the metals, and 27 eggshells had 3 or fewer metals present. Mean concentrations of metals were in the following order: Cu > Se> As> Pb > Cd Table 3. Arsenic was the most detected element in the sampled eggshells, with 92 percent of the eggshells containing arsenic. Cu had the highest mean concentration and range of all the metals, spanning from no detection to 8.88 ppm Table 3. On the other hand, only 26% of the eggshells had quantifiable cadmium concentrations, and 48% of the eggshells had concentrations below the detection limit. The metal concentrations we found are within the range of mean concentrations previously reported in eggshells of House Sparrows and other bird species (Fig 6, S1 Table). We found significant positive correlations between the metal pairs As and Cd (τ_a_ = 0.30, p < 0.001), and Cu and Pb (τ_a_ = 0.47, p < 0.001) Table 2, as well as a negative correlation between As and Pb (τ_a_ = −0.16, p = 0.02).

**Table 3:** Summary of eggshell metal concentrations.

n = 100	Ca (%)	As (µg/g)	Cd (µg/g)	Cu (µg/g)	Pb (µg/g)	Se (µg/g)
Geometric Mean	34.66	0.58	0.05	1.02	0.29	0.63
Arithmetic Mean (SD)	34.7 ± 1.62	0.72 ± 0.37	0.09 ± 0.15	1.97 ± 1.52	0.52 ± 0.62	0.87 ± 0.55
Median	34.95	0.77	0.05	2.06	0.41	0.83
Range (Min.-Max.)	30.90-37.50	ND-1.51	ND-0.99	ND-8.88	ND-4.66	ND-2.31
PQL limit	(<0.0005)	<0.30	<0.10	< 0.20	< 0.20	<0.30
Value Used	NA	0.225	0.075	NA	0.15	0.225
MDL limit	(<0.0001)	<0.15	<0.05	<0.10	<0.10	<0.15
Value Used	NA	0.075	0.025	0.05	0.05	0.075

The summary of the mass spectrometer results for the mean concentration of Ca, As, Cd, Cu, Pb and Se in the eggshell. For each metal the limitations of the testing equipment are defined by both the method detection limit (MDL), which indicates the lowest concentration the testing method can detect, and the practical quantification limit (PQL), which indicates the lowest concentration that can be reliably measured. In place of a null data point, half the MDL (value used) was substituted for eggshells where the concentrations of the metal were below detection. (ND- below detection limit).

### Results of model selection

Based on our model selection and averaging, PC1 and PC2 were not significant predictors of metal concentrations for any of the five metals Table 4. We did, however, find that the collection date was a positive predictor of Cu concentrations, indicating that eggs collected later in the breeding season had higher Cu concentrations. Additionally, metal concentrations were not a good predictor of complexity of eggshell spots Table 4. This is similar to previous research by Hargitai et al. indicating that spot intensity, spot size, spotting coverage, egg volume, and eggshell thickness were not related to concentrations of metals (Cu, Pb, and Zn) in the eggshell [22]. However, their research did find that eggs with more aggregated eggshell spotting distribution had higher concentrations of Cu in the eggshells, a variable not included in our study [22]. Other studies have shown that heavy metals impact eggshell coloration by altering UV reflectance, an eggshell characteristic that was not measured in this study [33,34].

**Table 4:** The averaged models for predicting the concentrations of metals and the spottiness of the egg from the model selection.

Metal	Parameter	Estimate	SE	Adjusted SE	Z-value	Probability
**Copper**	Intercept	1.974	0.149	0.151	13.081	<0.001
	Collection Date	0.355	0.161	0.163	2.181	0.029*
	PC2	−0.199	0.179	0.180	1.103	0.270
	Calcium	−0.071	0.131	0.132	0.538	0.591
	PC1	−0.053	0.121	0.122	0.439	0.660
	Collection Date:PC1	0.014	0.063	0.063	0.219	0.827
	Latitude	−0.019	0.074	0.075	0.250	0.802
**Cadmium**	Intercept	−1.123	0.270	0.273	4.109	<0.001
	Calcium	0.333	0.317	0.319	1.043	0.297
	PC1	0.394	0.290	0.292	1.349	0.177
	PC2	−0.510	0.353	0.356	1.434	0.157
	Thickness	−0.489	0.335	0.337	1.449	0.148
	Collection Date	−0.279	0.312	0.312	0.888	0.375
	Latitude	0.028	0.122	0.123	0.224	0.823
**Lead**	Intercept	0.524	0.061	0.062	8.444	<0.001
	PC1	−0.083	0.072	0.072	1.162	0.245
	Latitude	−0.011	0.036	0.036	0.313	0.754
	PC2	−0.006	0.028	0.028	0.204	0.838
**Arsenic**	Intercept	0.722	0.034	0.037	19.579	<0.001
	PC1	0.051	0.044	0.044	1.170	0.242
	Collection Date	−0.085	0.024	0.025	0.348	0.728
	Latitude	0.003	0.015	0.015	0.193	0.847
	PC2	−0.003	0.015	0.015	0.192	0.848
	Calcium	−0.0024	0.014	0.014	0.168	0.866
**Selenium**	Intercept	0.868	0.054	0.056	15.641	<0.001
	Latitude	0.100	0.066	0.067	1.497	0.134
	Calcium	0.018	0.042	0.042	0.427	0.670
	Longitude	−0.009	0.033	0.032	0.268	0.788
	PC1	−0.013	0.036	0.036	0.364	0.716
	Thickness	−0.002	0.019	0.019	0.127	0.899
**Spot Complexity**	Intercept	1.763	0.003	0.003	622.034	<0.001
	Copper	−0.003	0.003	0.003	1.115	0.265
	Lead	−0.003	0.003	0.003	1.071	0.284
	Selenium	−0.001	0.003	0.003	0.548	0.584

Full models can be seen in S2 and S3 Tables. PC2 is the loadings from the second principal component from the PCA results, PC1 is the loadings first principal component from the PCA results, Calcium is the calcium concentrations, Collection date:PC1 is an interaction term between collection date and the first principal component, SE = Standard error.

Our study found that trace elements varied across the collected eggshells. We found that essential metals (Cu and Se) were found at higher mean concentrations than the non-essential metals (Pb, As, Cd). We found mean Cu concentrations (1.97 ppm) to be higher than baseline concentrations of 1.65 ppm previously reported in House Sparrow eggshells [35]. In most studies, the mean concentration of Cu in the eggshell ranges from 0.5–2.0 ppm, placing our Cu concentrations in House Sparrow eggshells with range of most findings S1 Table. Like Cu, Se is another essential element that is toxic at high levels [36]. The concentration of Se we found in our House Sparrow eggshells (2.31 ppm) is comparable to the concentrations found in waterfowl and other species that feed on mostly aquatic organisms [23]. Fewer studies have tested the concentrations of metalloids, including Se and As, in eggshells. As far as we know, this is the first measurement of As in House Sparrow eggshells, and the mean concentration we found was comparable to concentrations found in other species in previous studies [37–39].

The mean Cd concentration (0.09 ppm) in the eggshells tested in this study was consistent with other studies that found low levels of Cd in eggshells Table 4. Most studies have reported concentrations less than 1.0 ppm, with mean concentrations as low as 0.002 ppm in Bridled Terns in Hong Kong [39]. Furness [40] argued that Cd is not excreted into the eggshells; instead, it is always present in the eggshells at low levels. However, more recent studies have found relatively high levels of Cd, including over 2.0 ppm in Eurasian Reed Warblers *Acrocephalus scirpaceus* in Poland [41] and 21.07 ppm in American Oystercatchers *Haematopus palliatus* in Argentina, suggesting that Cd is excreted into eggshells, but perhaps only when concentrations are high in the female [42]. Our Pb concentration (0.52 ppm) was within the range of the mean concentration of Pb found in other studies. Compared to other studies on House Sparrow eggshells, this concentration was lower than the 3.3 ppm mean concentration that Swaileh & Sansur [43] found in eggshells, but higher than the 0.42 ppm concentration found in eggshells from Baghdad, Iraq [44].

Additionally, we found significant positive correlations between individual pairs of metals (As-Cd and Cu-Pb) and a negative correlation between As and Pb. Swaileh & Sansur [43] also found a positive correlation between concentrations of Pb and Cu in House Sparrow eggshells. We are unaware of papers showing a significant relationship between As-Cd and As-Pb. Instead, previous research has shown a negative correlation between As and Se. In particular, the presence of As can reduce Se accumulation in the egg [3]. However, we did not find any significant correlations between As and Se in our study.

Despite being from a terrestrial, mostly granivorous bird, House Sparrow eggshells from across the United States had concentrations of metals and metalloids comparable to waterfowl and raptors that are on a higher trophic level. Previous work suggests that the level of contaminants in eggs is likely to reflect recent diet due to a rapid trophic transfer of nutrients before and during egg-laying [37]. While generally seed-eaters, there have been reported differences between the diet of House Sparrows in rural and urban areas, with urban House Sparrows consuming food from anthropogenic sources, which may alter both trophic level and exposure to dietary sources of contaminants [45,46]. In the case of House Sparrows, a non-migratory bird, the levels of metals in the egg are likely to reflect an area of a home range of up to 8 hectares, with House Sparrows spending most of their time occupying an area of less than 0.2 hectares [47]. This suggests that the eggshells with higher concentrations of metals are coming from areas of higher availability of the metals, as past studies have shown that birds from more contaminated sites had higher metal concentrations than those from control sites [37,39,48]. We are not able to determine if these concentrations in the eggshells are at levels that would impact the female bird or the embryo. However, the concentrations that we found in the eggshells are likely to be low compared to concentrations in other parts of the body. Swaileh & Sansur [43] found that out of 10 different areas of the body, eggshells contained the lowest metal richness. There have also been mixed studies comparing the concentrations found in eggshells to those in egg contents or body organs [49–50]. Metal concentrations in the embryo, vital organs and other parts of the House Sparrows may or may not be at toxic levels. Despite this uncertainty, these comparatively high levels when compared to eggshells of other bird species may make the widely distributed House Sparrows a valuable indicator of regions with unusually high concentrations of metals.

## Conclusions

Although House Sparrow eggshells could potentially be very important predictors, we found that eggshell characteristics did not reliably predict metal concentrations. Coloration was included in the averaged model for all five of the metals, but we found no significant relationship between color and metal concentrations. Speckling and calcium were included in most of the averaged models (missing from the averaged models for selenium and lead, respectively), but we also found no significant relationship. Instead of finding significant relationships between eggshell characteristics and metals, we found that collection date was a significant predictor of Cu concentrations, suggesting that Cu concentrations were higher in clutches laid later in the breeding season. Further research is needed to investigate the strength of this relationship and explore potential causes. Future research could explore dietary differences throughout the breeding seasons or potential links between Cu’s geographic distributions and climate’s impacts on breeding season [51–52].

Heavy metal concentrations were found in fairly high amounts in House Sparrow eggs across a large geographic range. While House Sparrow egg coloration varied greatly, we did not find any connection between eggshell heavy metal concentrations and eggshell coloration. Our findings indicate that there could be separate research into using House Sparrow eggs as bioindicators of heavy metals and exploring eggshell coloration in House Sparrows.

## Supporting information

S1 Table Mean concentrations and standard deviation of metals found in eggshells in previous studies. Previous studies have found varying mean concentration of Ca, As, Cd, Cu, Pb, and Se in eggshells from varying species. Bolded values are reported mean concentrations higher than those found in this present study. Unbolded values are mean concentrations lower than those found in this present study. * indicates median reported instead of mean [53–66].
(DOCX)

S2 Table Abbreviations for variables in S3 Table. The following supplemental table (S3 Table) provides a comprehensive list of models used in the averaged model in Table 4. The abbreviations in this table (S2 Table) are used to represent each parameter in the model.
(DOCX)

S3 Table Models with a ΔAIC_c_ less than 2.0. The models included in this table had a ΔAIC_c_ less than 2.0. These models were used to calculate the averaged model in Table 4. A list of abbreviations used in the table are in S2 Table.
(DOCX)

## References

[1] Becker PH. Biomonitoring with birds. In: Markert BA, Breure AM, Zechmeister HG, editors. Bioindicators and biomonitors. Oxford: Elsevier Science; 2003. p. 677–736.

[2] Burger J. Heavy metals in avian eggshells: another excretion method. J Toxicol Environ Health. 1994;41(2):207–20. doi: 10.1080/15287399409531837 8301699

[3] Stanley TR, Spann JW, Smith GJ, Rosscoe R. Main and interactive effects of As and selenium on mallard reproduction and duckling growth and survival. Arch Environ Contam Toxicol. 1994;26:444–51.

[4] Eeva T, Lehikoinen E. Rich calcium availability diminishes heavy metal toxicity in Pied Flycatcher. Funct Ecol. 2004;18:548–53.

[5] Ayaş Z, Celikkan H, Aksu ML. Lead (Pb) and copper (Cu) concentration in the eggshells of Audouin’s *gull (Larus audouinii)* in Turkey. Turk Zool Derg. 2008;32:379–84.

[6] Morera M, Sanpera C, Crespo S, Jover L, Ruiz X. Inter- and intraclutch variability in heavy metals and selenium levels in Audouin’s gull eggs from the Ebro delta, Spain. Arch Environ Contam Toxicol. 1997;33:71–5.

[7] Bumpus HC. The variations and mutations of the introduced sparrow: *Passer domesticus*. Boston: Ginn and Company; 1896.

[8] López de Hierro MDG, de Neve L. Pigment limitation and female reproductive characteristics influence eggshell spottiness and ground colour variation in the House Sparrow (*Passer domesticu*s). J Ornithol. 2010;151:833–40.

[9] 16 U.S.C. 703-712; Ch. 128. 40 Stat. 755. July 3, 1918.

[10] Ravinet M, Elgvin TO, Trier C, Aliabadian M, Gavrilov A, Sætre GP. Signatures of human commensalism in the House Sparrow genome. Proc Biol Sci. 2018;285:1884. 10.1098/rspb.2018.1246 PMC6111181 30089626

[11] Jagannath A, Shore RF, Walker LA, Ferns PN, Gosler AG. Eggshell pigmentation indicates pesticide contamination. J Appl Ecol. 2008;45:133–40.

[12] Hanley D, Doucet SM. Does environmental contamination influence egg coloration? A long-term study in Herring Gulls. J Appl Ecol. 2012;49:1055–63.

[13] Casini S, Fossi MC, Leonzio C, Renzoni A. Review: porphyrins as biomarkers for hazard assessment of bird populations: destructive and non-destructive use. Ecotoxicology. 2003;12(1–4):297–305. doi: 10.1023/a:1022519214505 12739876

[14] Mateo R, Castells G, Green AJ, Godoy C, Cristòfol C. Determination of porphyrins and biliverdin in bile and excreta of birds by a single liquid chromatography-ultraviolet detection analysis. J Chromatogr B Analyt Technol Biomed Life Sci. 2004;810(2):305–11. doi: 10.1016/j.jchromb.2004.08.019 15380729

[15] Hanley D, Grim T, Cassey P, Hauber ME. Not so colourful after all: eggshell pigments constrain avian eggshell colour space. Biol Lett. 2015;11(5):20150087. doi: 10.1098/rsbl.2015.0087 25994009 PMC4455735

[16] Kennedy GY, Vevers HG. A survey of avian eggshell pigments. Comp Biochem Physiol B. 1976;55(1):117–23. doi: 10.1016/0305-0491(76)90183-8 947658

[17] Hamchand R, Hanley D, Prum RO, Brückner C. Expanding the eggshell colour gamut: uroerythrin and bilirubin from tinamou (Tinamidae) eggshells. Sci Rep. 2020;10(1):11264. doi: 10.1038/s41598-020-68070-7 32647200 PMC7347609

[18] Gosler AG, Higham JP, James Reynolds S. Why are birds’ eggs speckled?. Ecology Letters. 2005;8(10):1105–13. doi: 10.1111/j.1461-0248.2005.00816.x

[19] Solomon SE. Egg shell pigmentation. In: Wells RG, Belyarin CG, editors. Egg quality-current problems and recent advances. London: Butterworths; 1987. p. 147–57.

[20] Rodriguez-Navarro AB, Gaines KF, Romanek CS, Masson GR. Mineralization of clapper rail eggshell from a contaminated salt marsh system. Arch Environ Contam Toxicol. 2002;43(4):449–60. doi: 10.1007/s00244-002-0266-8 12399916

[21] Gosler AG, Wilkin TA. Eggshell speckling in a passerine bird reveals chronic long-term decline in soil calcium. Bird Study. 2017;64(2):195–204. doi: 10.1080/00063657.2017.1314448

[22] Hargitai R, Nagy G, Nyiri Z, Bervoets L, Eke Z, Eens M, et al. Effects of breeding habitat (woodland versus urban) and metal pollution on the egg characteristics of great tits (*Parus major*). Sci Total Environ. 2016;544:31–8. doi: 10.1016/j.scitotenv.2015.11.116 26657247

[23] Beyer WN, Heinz GH, Redmon-Norwood AW. Selenium in birds. Environmental Contaminants in Wildlife: Interpreting Tissue Concentrations. Boca Raton: CRC Press; 1996. p. 447–58.

[24] Davis JM, Otto DA, Weil DE, Grant LD. The comparative developmental neurotoxicity of lead in humans and animals. Neurotoxicol Teratol. 1990;12(3):215–29. doi: 10.1016/0892-0362(90)90093-r 2196421

[25] Khan A, Iftikhar H, Adeel H, Muhammad S, Khan Z, Rao ZA. Toxico-pathological aspects of arsenic in birds and mammals: A review. Int J Agric Biol. 2014;16:1213–24.

[26] Gómez J, Liñán‐Cembrano G. SpotEgg: an image‐processing tool for automatised analysis of colouration and spottiness. Journal of Avian Biology. 2016;48(4):502–12. doi: 10.1111/jav.01117

[27] Coffin D. DCRAW application. 2015. https://www.cybercom.net/~dcoffin/dcraw/

[28] Revelle W. Psych: Procedures for personality and psychological research. Evanston, Illinois, USA: Northwestern University; 2015. http://CRAN.R-project.org/package=psychVersion=1.5.8

[29] Igic B, Hauber ME, Galbraith JA, Grim T, Dearborn DC, Brennan PLR, et al. Comparison of micrometer- and scanning electron microscope-based measurements of avian eggshell thickness. Journal of Field Ornithology. 2010;81(4):402–10. doi: 10.1111/j.1557-9263.2010.00296.x

[30] Orłowski G, Merta D, Pokorny P, Łukaszewicz E, Dobicki W, Kobielski J, et al. Eggshell resorption, and embryonic mobilization and accumulation of calcium and metals in eggs of wild and captive Capercaillies *Tetrao urogallus*. Environ Pollut. 2019;249:152–62. doi: 10.1016/j.envpol.2019.03.010 30884394

[31] R CT. R: A language and environment for statistical computing. Vienna, Austria: R Foundation for Statistical Computing; 2019. http://www.R-project.org/

[32] Barton K. Mu-MIn: Multi-model inference. R Package Version 0.12.2/r18. 2009. http://R-Forge.R-project.org/projects/mumin/

[33] Mari L, Šulc M, Szala K, Troscianko J, Eeva T, Ruuskanen S. Heavy metal pollution exposure affects egg coloration but not male provisioning effort in the pied flycatcher *Ficedula hypoleuca*. J Avian Biol. 2025. doi: e03283

[34] Martínez A, López-Rull I. Metals Can Change the Colors of Eggshells but How Is This Related to Oxidative Stress and Antibacterial Capacity?. ACS Omega. 2024;9(5):5601–7. doi: 10.1021/acsomega.3c07702 38343943 PMC10851376

[35] Anderson TR. Biology of the ubiquitous house sparrow: from genes to populations. New York: Oxford University Press; 2006.

[36] Lemly AD. Ecosystem recovery following selenium contamination in a freshwater reservoir. Ecotoxicol Environ Saf. 1997;36(3):275–81. doi: 10.1006/eesa.1996.1515 9143456

[37] Ruuskanen S, Laaksonen T, Morales J, Moreno J, Mateo R, Belskii E, et al. Large-scale geographical variation in eggshell metal and calcium content in a passerine bird (*Ficedula hypoleuca*). Environ Sci Pollut Res Int. 2014;21(5):3304–17. doi: 10.1007/s11356-013-2299-0 24234761

[38] Jakubas D, Kitowski I, Wiącek D, Bzoma S. Inter-species and inter-colony differences in elemental concentrations in eggshells of sympatrically nesting great cormorants *Phalacrocorax carbo* and grey herons *Ardea cinerea*. Environ Sci Pollut Res Int. 2019;26(3):2747–60. doi: 10.1007/s11356-018-3765-5 30484052 PMC6338717

[39] Lam JCW, Tanabe S, Lam MHW, Lam PKS. Risk to breeding success of waterbirds by contaminants in Hong Kong: evidence from trace elements in eggs. Environ Pollut. 2005;135(3):481–90. doi: 10.1016/j.envpol.2004.11.021 15749545

[40] Beyer WN, Heinz GH, Redmon-Norwood AW. Cadmium in birds. Environmental contaminants in wildlife: interpreting tissue concentrations. Boca Raton: CRC Press; 1996. p. 389–404.

[41] Orłowski G, Hałupka L, Pokorny P, Klimczuk E, Sztwiertnia H, Dobicki W. The effect of embryonic development on metal and calcium content in eggs and eggshells in a small passerine. Ibis. 2015;158(1):144–54. doi: 10.1111/ibi.12327

[42] Simonetti P, Botté SE, Marcovecchio JE. Exceptionally high Cd levels and other trace elements in eggshells of American oystercatcher (*Haematopus palliatus*) from the Bahía Blanca Estuary, Argentina. Mar Pollut Bull. 2015;100(1):495–500. doi: 10.1016/j.marpolbul.2015.09.006 26362457

[43] Swaileh KM, Sansur R. Monitoring urban heavy metal pollution using the House Sparrow (*Passer domesticus*). J Environ Monit. 2006;8(1):209–13. doi: 10.1039/b510635d 16395481

[44] Al-Obaidi FA, Mehdi BI, Shadeedi SMA. Identification of inorganic elements in eggshell of some wild birds in Baghdad. Adv Appl Sci Research. 2012;3:452–1458.

[45] Martin LBI, Fitzgerald L. A taste for novelty in invading house sparrows, *passer domesticus*. Behav Ecol. 2005;16:702–7.

[46] Gavett AP, Wakeley JS. Diets of House Sparrows in Urban and Rural Habitats. Wilson Bulletin. 1986;98:137–44.

[47] Vangestel C, Braeckman BP, Matheve H, Lens LUC. Constraints on home range behaviour affect nutritional condition in urban house sparrows (*Passer domesticus*). Biol J Linn Soc Lond. 2010;101:41–50.

[48] Ayaş Z. Trace element residues in eggshells of grey heron (*Ardea cinerea*) and black-crowned night heron (*Nycticorax nycticorax*) from Nallihan Bird Paradise, Ankara-Turkey. Ecotoxicology. 2007;16(4):347–52. doi: 10.1007/s10646-007-0132-6 17364239

[49] Dauwe T, Bervoets L, Blust R, Pinxten R. Are eggshells and egg contents of great and blue tits suitable as indicators of heavy metal pollution?. Belg J of Zool. 1999;129:439–47.

[50] Agusa T, Matsumoto T, Ikemoto T, Anan Y, Kubota R, Yasunaga G, et al. Body distribution of trace elements in black-tailed gulls from Rishiri Island, Japan: age-dependent accumulation and transfer to feathers and eggs. Environ Toxicol Chem. 2005;24(9):2107–20. doi: 10.1897/04-617r.1 16193736

[51] Hunter BA, Johnson MA, Thompson DJ. Ecotoxicology of copper and cadmium in a contaminated grassland ecosystem. III. Small mammals. J Appl Ecol. 1987;24:601–14.

[52] Smith DB, Cannon WF, Woodruff LG, Solano F, Ellefsen KJ. Geochemical and mineralogical maps for soils of the conterminous United States. USGS Open-File Report. 2014;1082:1–386.

[53] Ding J, Yang W, Yang Y, Ai S, Bai X, Zhang Y. Variations in tree sparrow (*Passer montanus*) egg characteristics under environmental metal pollution. Sci Total Environ. 2019;687:946–55. doi: 10.1016/j.scitotenv.2019.06.140 31412498

[54] Kraus ML. Bioaccumulation of heavy metals in pre-fledgling tree swallows, *Tachycineta bicolor*. Bull Environ Contam Toxicol. 1989;43(3):407–14. doi: 10.1007/BF01701876 2790248

[55] Mora MA. Heavy metals and metalloids in egg contents and eggshells of passerine birds from Arizona. Environ Pollut. 2003;125(3):393–400. doi: 10.1016/s0269-7491(03)00108-8 12826417

[56] Orlowski G, Kasprzykowski Z, Dobicki W, Pokorny P, Przemyslaw P, Polechoński R. Geographical and habitat differences in concentrations of copper, zinc and arsenic in eggshells of the Rook *Corvus frugilegus* in Poland. J Ornithol. 2010;151:279–86.

[57] Orłowski G, Kasprzykowski Z, Dobicki W, Pokorny P, Wuczyński A, Polechoński R, et al. Residues of chromium, nickel, cadmium and lead in Rook *Corvus frugilegus* eggshells from urban and rural areas of Poland. Sci Total Environ. 2014;490:1057–64. doi: 10.1016/j.scitotenv.2014.05.105 24914534

[58] Ashkoo A, Amininasab SM, Zamani-Ahmadmahmoodi R. Bioaccumulation of heavy metals in eggshell and egg content of seabirds: Lesser (*Thalasseus bengalensis*) and Greater Crested Tern (*Thalasseus bergii*). Mar Pollut Bull. 2020;154:111126. doi: 10.1016/j.marpolbul.2020.111126 32319936

[59] Currie D, Valkama J. Limited effects of heavy metal pollution on foraging and breeding success in the curlew (*Numenius arquata*). Environ Pollut. 1998;101(2):253–61. doi: 10.1016/s0269-7491(98)00037-2 15093087

[60] Dev B, Gupta A, Bhattacharjee PC. Heavy metals in egg shells of six species of Ardeidae (Aves) from Barak Valley, Assam. Assam Uni J Sci Technol. 2010;5:48–52.

[61] Dolci NN, Sá F, da Costa Machado E, Krul R, Rodrigues Neto R. Trace elements in feathers and eggshells of brown booby *Sula leucogaster* in the Marine National Park of Currais Islands, Brazil. Environ Monit Assess. 2017;189(10):496. doi: 10.1007/s10661-017-6190-1 28891020

[62] Hashmi MZ, Malik RN, Shahbaz M. Heavy metals in eggshells of cattle egret (*Bubulcus ibis*) and little egret (*Egretta garzetta*) from the Punjab province, Pakistan. Ecotoxicol Environ Saf. 2013;89:158–65. doi: 10.1016/j.ecoenv.2012.11.029 23260238

[63] Ikemoto T, Kunito T, Tanabe S, Tsurumi M, Sato F, Oka N. Non-destructive monitoring of trace element levels in short-tailed albatrosses (*Phoebastria albatrus*) and black-footed albatrosses (*Phoebastria nigripes*) from Torishima Island, Japan using eggs and blood. Mar Pollut Bull. 2005;51(8–12):889–95. doi: 10.1016/j.marpolbul.2005.06.003 16023681

[64] Kim J, Oh J-M. Trace element concentrations in eggshells and egg contents of black-tailed gull (*Larus crassirostris*) from Korea. Ecotoxicology. 2014;23(7):1147–52. doi: 10.1007/s10646-014-1256-0 24859774

[65] Metcheva R, Yurukova L, Teodorova SE. Biogenic and toxic elements in feathers, eggs, and excreta of Gentoo penguin (*Pygoscelis papua ellsworthii*) in the Antarctic. Environ Monit Assess. 2011;182(1–4):571–85. doi: 10.1007/s10661-011-1898-9 21340549

[66] Rickard WH, Schuler CA. Mineral Composition of Eggshells of Wild Birds from the Columbia Basin, Washington. Northwestern Naturalist. 1990;71(1):5. doi: 10.2307/3536546

