# Supplementary material for: Heavy metals and eggshell coloration in House Sparrow (Passer domesticus) eggshells across the Eastern United States
Source: PLoS One. 2026 Feb 25;21(2):e0336122. doi: 10.1371/journal.pone.0336122 (PMC12935262; doi:10.1371/journal.pone.0336122)
Supplement: S1 Table — Previous studies have found varying mean concentration of Ca, As, Cd, Cu, Pb, and Se in eggshells from varying species. Bolded values are reported mean concentrations higher than those found in this present study. Unbolded values are mean concentrations lower than those found in this present study. * indicates median reported instead of mean [53–66]. (DOCX) [file pone.0336122.s001.docx]

**S1 Table. Mean concentrations and standard deviation of metals found in eggshells in previous studies.**

| **Study** | **Location** | **Species** | **Ca** | **As** | **Cd** | **Cu** | **Pb** | **Se** |
| --- | --- | --- | --- | --- | --- | --- | --- | --- |
| ***House Sparrow*** |  |  |  |  |  |  |  |  |
| Present Study | USA | House Sparrow | 34.7±1.62 | 0.72±0.37 | 0.09±0.15 | 1.97±1.52 | 0.52±0.62 | 0.87±0.55 |
| Swaileh & Sansur 2006 [44] | West Bank | House Sparrow | **-** | **-** | 0.01±0.00 | 1.00±0.10 | **3.3±0.6** | **-** |
| Al-Obaidi et al. 2011 [45] | Baghdad, Iraq | House Sparrow | 97.30±0.85 | **-** | **-** | **-** | 0.41±0.02 | **-** |
|  |  | Collared Dove | 97.80±0.86 | **-** | **-** | **-** | 0.42±0.02 | **-** |
|  |  | Rock Dove | 97.80±0.86 | **-** | **-** | **-** | 0.40±0.03 | **-** |
|  |  | White-eared Bulbul | 97.40±0.84 | **-** | **-** | **-** | 0.44±0.04 | **-** |
| ***Other Passerines and Terrestrial Birds*** | |  |  |  |  |  |  |  |
| Dauwe et al. 1999 [49] | Antwerp, Belgium | Great and Blue Tits | **-** | **1.20±0.60** | 0.05±0.01 | 1.72±0.23 | 0.37±0.16 | **-** |
|  | Hoboken, Belgium | Great and Blue Tits | **-** | **4.20±0.80** | **0.80±0.60** | **3.20±0.50** | **15.00±4.00** | **-** |
| Ding et al. 2019 [53] | Baiyin, China | Tree Sparrow | **-** | **-** | **-** | **5.16±2.45** | **49.15±15.39** | **-** |
|  | Liujiaxia, China | Tree Sparrow | **-** | **-** | **-** | **3.74±2.05** | **39.94±2.76** | **-** |
| Hargitai et al. 2016 [23] | Budapest, Hungary | Great Tits | **-** | **-** | **-** | 1.38±0.37 | 0.18±0.06 | **-** |
|  | Pilis Mountains, Hungary | Great Tits | **-** | **-** | **-** | 1.2±0.22 | 0.11±0.05 | **-** |
| Kraus 1989 [54] | New Jersey, USA | Tree Swallows | **-** | **-** | **1.8** | **2.4±2.5** | **90.9±59.5** | **-** |
| Mora et al. 2003 [55} | Arizona, USA | Willow Flycatcher | **-** | **1.30±0.20** | **-** | **2.50±0.90** | **0.90±0.60** | **1.20±0.70** |
|  |  | Yellow-breasted Chat | **-** | **2.10±0.40** | **-** | **6.20±8.00** | **0.60±0.70** | 0.50±0.30 |
| Orlowski et al. 2010 [56] | Poland | Rook | **-** | **32.57** | **-** | **8.13** | **-** | **-** |
| Orlowski et al. 2014 [57] | Poland | Rook | **-** | **-** | **0.51** | **-** | **3.29** | **-** |
| Orlowski et al. 2015 [42] | Milicz Ponds, Poland | Reed Warbler (no embryo) | 25.36 | **-** | **2.1** | **7.91** | **5.65** | **-** |
|  |  | Reed Warbler (embryo) | 23.13 | **-** | **2.36** | **9.69** | **7.12** | **-** |
| Ruuskanen et al. 2014 [38] | Europe & Russia | European Pied Flycatcher | 27.62-33.58 | .008-1.32 | **-** | **2.19-2.83** | 0.17-0.45 | **-** |
| ***Waterbirds and Raptors*** |  |  |  |  |  |  |  |  |
| Agusa et al. 2005 [50] | Rishiri Island, Japan | Black-tailed Gull | **-** | **-** | 0.01±0.01 | 0.54±0.09 | 0.06±0.04 | 0.42±0.15 |
| Ashkoo et al. 2020 [58] | Nakhiloo Island, Iran | Greater Crested Tern | **-** | **-** | **4.15±1.31** | **-** | **2.48±1.77** | **-** |
|  |  | Lesser Crested Tern | **-** | **-** | **3.13±1.62** | **-** | **2.8±1.14** | **-** |
| Ayaş et al. 2007 [48] | Anakara, Turkey | Black-crowned night heron | **-** | **-** | **0.23±0.19*** | 1.69±0.17* | **1.11±0.87*** | **-** |
|  |  | Grey Heron | **-** | **-** | **0.93±0.49*** | **6.76±1.20*** | **6.83±2.75*** | **-** |
| Ayaş et al. 2008 [5] | Aydinick Island, Turkey | Audouin's gull | **-** | **-** | **-** | 1.86±2.57 | **0.95±1.01** | **-** |
|  | Karaburun Island, Turkey | Audouin's gull | **-** | **-** | **-** | **10.2±16.04** | **4.60±5.81** | **-** |
| Burger 1994 [2] | Long Island, USA | Herring Gull | **-** | **-** | 0.05±0.01 | **-** | 0.30±0.05 | 0.40±0.03 |
|  |  | Roseate Tern | **-** | **-** | **0.10±0.04** | **-** | **1.20±0.30** | <0.005 |
| Currie and Valkama 1997 [59] | Harjavalta, Finland | Numenius arquata | 0.24±0.03 | **-** | **-** | **9.56±0.61** | **-** | **-** |
|  | Kauhava, Finland | Numenius arquata | 0.30±0.05 | **-** | **-** | **8.03±0.24** | **-** | **-** |
|  | Vammala, Finland | Numenius arquata | 0.30±0.05 | **-** | **-** | **7.70±0.28** | **-** | **-** |
| Dev et al. 2010 [60] | Assam India | Ardeola grayii | **-** | **-** | 0.08 | **-** | **0.91** | **-** |
|  |  | Boturus Stellaris | **-** | **-** | 0.05 | **-** | **0.79** | **-** |
|  |  | Bubulcus ibis | **-** | **-** | 0.06 | **-** | **0.58** | **-** |
|  |  | Egretta garzetta | **-** | **-** | 0.06 | **-** | **0.81** | **-** |
|  |  | Ixobrychus cinnamomeus | **-** | **-** | 0.07 | **-** | **0.84** | **-** |
|  |  | Ixobrychus minutus | **-** | **-** | 0.07 | **-** | **0.81** | **-** |
| Dolci et al. 2017 [61] | Currais Island, Brazil | Brown Booby | **-** | **2.37±1.01** | 0.03±0.03 | 0.99±0.48 | **-** | **-** |
| Hashmi et al. 2013 [62] | Pakistan | Cattle Egret | **-** | **-** | **0.10-1.23** | 0.06-0.11 | 0.13-5.40 | **-** |
|  |  | Little Egret | **-** | **-** | **0.75-1.02** | 0.12-0.29 | **1.09-1.90** | **-** |
| Ikemoto et al. 2005 [63] | Tarishima Island, Japan | Black-footed albatross | **-** | **-** | **0.10±0.35** | 0.78±0.13 | 0.04±0.03 | 0.15±0.06 |
|  |  | Short-Tailed albatross | **-** | **-** | 0.01±0.01 | 0.77±0.10 | 0.01±0.006 | 0.08±0.03 |
| Jakubas et al. 2019 [39] | Poland | Great Cormorants | 34.3* | 0.42* | 0.01* | 1.46* | **0.54*** | 0.73* |
|  | Poland | Grey Heron | 27.6* | 0.11* | 0.02* | 0.81* | 0.43* | 0.7* |
| Kim and Oh 2014 [64] | Korea | Black Tailed Gull | **-** | **-** | **0.45±0.28** | **2.80±0.92** | **3.10±1.35** | **-** |
| Lam et al. 2005 [40] | Hong Kong | Black-crowned Night Heron | **-** | **-** | 0.01±0.002 | 1.12±0.40 | 0.03±0.01 | **8.16±0.20** |
|  |  | Bridled Tern | **-** | 0.40±0.07 | 0.002±0.001 | 1.24±0.41 | 0.06±0.04 | **15.58±1.87** |
|  |  | Little Egret | **-** | **-** | 0.006±0.002 | 1.60±0.73 | 0.15±0.17 | **7.59±0.67** |
| Metcheva et al. 2011 [65] | Anatartica | Gentoo Penguin | **17.04±4.75** | **<0.3** | <0.05 | 1.24±0.40 | 0.68±0.30 | **<0.05** |
| Morera et al. 1997 [6] | Ebro Delta, Spain | Audouin's Gull | **-** | **-** | - | 2.14±0.70 | - | **4.12±1.45** |
| Rickard and Schuler 1990 [66] | Washington, USA | Canada Goose | **37.8** | **-** | - | 12.2 | - | **-** |
|  |  | Bald Eagle | **36.4** | **-** | - | 12 | - | **-** |
|  |  | Ferruginous Hawk | **37.2** | **-** | - | 9.7 | - | **-** |
|  |  | Golden Eagle | **36** | **-** | - | 10 | - | **-** |
|  |  | Great Blue Heron | **37.3** | **-** | - | 9 | - | **-** |
|  |  | Osprey | **35.7** | **-** | - | 9.3 | - | **-** |
|  |  | Ring-billed Gull | **36.4** | **-** | - | 8.5 | - | **-** |
|  |  | Swainson's Hawk | **32.7** | **-** | - | 8.5 | - | **-** |
| Rodriquez-Navarro et al. 2002 [21] | Blythe Island, GA, USA | Clapper Rails | **-** | **0.21±0.09** | - | 1.37±0.64 | 0.23±0.10 | **0.71±0.28** |
|  | Brunswick, GA, USA | Clapper Rails | **-** | **0.21±0.09** | - | 1.71±0.58 | 0.37±0.52 | **0.90±0.26** |
| Simonetti et al. 2015 [43] | Bahia Blanca, Argentina | American Oystercatcher | **-** | **-** | 13.28 | 2.02 | 7.23 | **-** |

Previous studies have found varying mean concentrations of Ca, As, Cd, Cu, Pb, and Se in eggshells from varying species. Bolded values are reported mean concentrations higher than those found in this present study. Unbolded values are mean concentrations lower than those found in this present study. * indicates median reported instead of mean.
