# Supplementary material for: Heavy metals and eggshell coloration in House Sparrow (Passer domesticus) eggshells across the Eastern United States
Source: PLoS One. 2026 Feb 25;21(2):e0336122. doi: 10.1371/journal.pone.0336122 (PMC12935262; doi:10.1371/journal.pone.0336122)
Supplement: S2 Table — The following supplemental table (S3 Table) provides a comprehensive list of models used in the averaged model in Table 4. The abbreviations in this table (S2 Table) are used to represent each parameter in the model. (DOCX) [file pone.0336122.s002.docx]

**S2 Table. Abbreviations for variables in S3 Table.**

| **Variable** | **Abbreviation** |
| --- | --- |
| Intercept | I |
| Calcium | Ca |
| PC1 | P1 |
| PC2 | P2 |
| Thickness | T |
| Collection Date | D |
| Latitude | LA |
| Collection Date:PC1 | D:P1 |
| Longitude | LO |
| Copper | Cu |
| Selenium | Se |
| Lead | Pb |
| Cadmium | Cd |
| Arsenic | As |

The following supplemental table (S3 Table) provides a comprehensive list of models used in the averaged model in Table 3. The abbreviations in this table (S2 Table) are used to represent each parameter in the model.
