# Supplementary material for: Heavy metals and eggshell coloration in House Sparrow (Passer domesticus) eggshells across the Eastern United States
Source: PLoS One. 2026 Feb 25;21(2):e0336122. doi: 10.1371/journal.pone.0336122 (PMC12935262; doi:10.1371/journal.pone.0336122)
Supplement: S3 Table — The models included in this table had a ΔAICc less than 2.0. These models were used to calculate the averaged model in Table 4. A list of abbreviations used in the table are in S2 Table. (DOCX) [file pone.0336122.s003.docx]

**S3 Table.** Models with a ΔAIC_c_ less than 2.0.

| Metal | Model | *df* | AIC_c_ | ΔAIC_c_ | *w* | R |
| --- | --- | --- | --- | --- | --- | --- |
| Copper | I + D + P2 | 4 | 366.89 | 0.00 | 0.047 | 0.07 |
|  | I + D + P2 + Ca | 5 | 367.36 | 0.47 | 0.037 | 0.09 |
|  | I + D | 3 | 367.70 | 0.81 | 0.031 | 0.05 |
|  | I + D + P2 + P1 | 5 | 367.75 | 0.86 | 0.031 | 0.09 |
|  | I + D + Ca | 4 | 368.48 | 1.59 | 0.021 | 0.06 |
|  | I + D + P1 | 6 | 368.50 | 1.61 | 0.021 | 0.10 |
|  | I + D + P2 + Ca + LA | 4 | 368.58 | 1.69 | 0.020 | 0.06 |
|  | I + D + P2 + Ca + P1 | 6 | 368.59 | 1.70 | 0.020 | 0.10 |
|  | I + D + P2 + Ca + LA | 6 | 368.61 | 1.72 | 0.020 | 0.10 |
|  | I + D + P2 + LA | 5 | 366.64 | 1.75 | 0.020 | 0.08 |
| Cadmium | I + Ca + P1 + P2 + T | 5 | 112.18 | 0 | 0.050 | 0.17 |
|  | I + Ca + P1 + P2 + T + D | 6 | 112.46 | 0.28 | 0.043 | 0.19 |
|  | I + Ca + P2 + T + D | 5 | 112.93 | 0.75 | 0.034 | 0.17 |
|  | I + P1 + P2 + T | 4 | 113.07 | 0.89 | 0.032 | 0.15 |
|  | I + P1 + P2 + T +D | 5 | 113.08 | 0.90 | 0.032 | 0.16 |
|  | I + Ca + P1 + P2 | 4 | 113.48 | 1.30 | 0.026 | 0.14 |
|  | I + Ca + P1 + T +D | 5 | 113.82 | 1.64 | 0.022 | 0.16 |
|  | I + Ca + P1 + P2 + T + LA | 6 | 113.91 | 1.73 | 0.021 | 0.18 |
|  | I + Ca + P1 + P2 + T +D + LA | 7 | 113.92 | 1.74 | 0.021 | 0.20 |
|  | I + P1 + T + D | 4 | 113.98 | 1.80 | 0.020 | 0.14 |
|  | I + P2 + T +D | 4 | 114.01 | 1.83 | 0.020 | 0.14 |
|  | I + Ca + P1 + P2 +D | 5 | 114.03 | 1.85 | 0.020 | 0.16 |
| Lead | I + P1 | 3 | 189.30 | 0.00 | 0.083 | 0.03 |
|  | I | 2 | 190.28 | 0.98 | 0.051 | 0.00 |
|  | I + P1 + LA | 4 | 190.64 | 1.34 | 0.042 | 0.04 |
|  | I + P1 + PC2 | 4 | 191.12 | 1.82 | 0.033 | 0.03 |
| Arsenic | I + P1 | 3 | 85.20 | 0.00 | 0.074 | 0.04 |
|  | I | 2 | 86.65 | 1.45 | 0.036 | 0.00 |
|  | I + P1 + D | 4 | 86.84 | 1.64 | 0.033 | 0.04 |
|  | I + P1 + LA | 4 | 86.92 | 1.72 | 0.031 | 0.04 |
|  | I + P1 + P2 | 4 | 86.92 | 1.72 | 0.031 | 0.04 |
|  | I + P1 +Ca | 4 | 87.03 | 1.83 | 0.030 | 0.04 |
|  | I + D | 5 | 87.12 | 1.91 | 0.028 | 0.02 |
| Selenium | I + LA | 3 | 167.51 | 0.00 | 0.061 | 0.04 |
|  | I + LA + Ca | 4 | 168.36 | 0.85 | 0.040 | 0.05 |
|  | I + LA + LO | 4 | 168.69 | 1.18 | 0.034 | 0.05 |
|  | I + LA + P1 | 4 | 168.77 | 1.26 | 0.032 | 0.04 |
|  | I | 2 | 169.04 | 1.53 | 0.028 | 0.00 |
|  | I + LA + Ca + P1 | 5 | 169.31 | 1.8 | 0.025 | 0.06 |
|  | I + LA + T | 4 | 169.47 | 1.96 | 0.023 | 0.04 |
| Fractal | I | 2 | -427.91 | 0.00 | 0.113 | 0.00 |
| Dimension | I + Cu | 3 | -427.62 | 0.31 | 0.097 | 0.02 |
|  | I + Pb | 3 | -427.53 | 0.39 | 0.093 | 0.02 |
|  | I + Se | 3 | -426.11 | 1.81 | 0.046 | 0.03 |
|  | I + Cu + Pb | 4 | -425.99 | 1.93 | 0.043 | 0.02 |

The models included in this table had a ΔAIC_c_ less than 2.0. These models were used to calculate the averaged model in Table 3. A list of abbreviations used in the table are in S2 Table.
